# Game Theoretical Analysis on Cooperation Stability and Incentive Effectiveness in Community Networks

**DOI:** 10.1371/journal.pone.0141755

**Published:** 2015-11-09

**Authors:** Kaida Song, Rui Wang, Yi Liu, Depei Qian, Han Zhang, Jihong Cai

**Affiliations:** 1 State Key Laboratory of Software Development Environment, Beihang University, Beijing, China; 2 School of Computer Science and Engineering, Beihang University, Beijing, China; 3 Science and Technology on Special System simulation Laboratory, Beijing Simulation Center, Beijing, China; Kyushu University, JAPAN

## Abstract

Community networks, the distinguishing feature of which is membership admittance, appear on P2P networks, social networks, and conventional Web networks. Joining the network costs money, time or network bandwidth, but the individuals get access to special resources owned by the community in return. The prosperity and stability of the community are determined by both the policy of admittance and the attraction of the privileges gained by joining. However, some misbehaving users can get the dedicated resources with some illicit and low-cost approaches, which introduce instability into the community, a phenomenon that will destroy the membership policy. In this paper, we analyze on the stability using game theory on such a phenomenon. We propose a game-theoretical model of stability analysis in community networks and provide conditions for a stable community. We then extend the model to analyze the effectiveness of different incentive policies, which could be used when the community cannot maintain its members in certain situations. Then we verify those models through a simulation. Finally, we discuss several ways to promote community network’s stability by adjusting the network’s properties and give some proposal on the designs of these types of networks from the points of game theory and stability.

## Introduction

Compared to fully-opened networks, community networks provide the possibility to maintain stable cooperation on shared resources among different members [1], which is the prerequisite of Peer-to-peer (P2P) networks. Unfortunately, maintaining stable cooperation is extremely difficult over the Internet. The difficulty in forming long-term cooperation among network nodes over the Internet exists due to a lack of restriction among these nodes. Due to the contract with the community, it is easy to establish trust and repay in a community environment that could attract users to return after they have left the community. Although almost every community network provides an incentive policy, which determines the lifetime of the community, those policies don't always work well.

Therefore, enhancing the cooperation with a feasible policy determines the success of the community network. Plenty of research has been done on cooperation analysis in the P2P network environment [2, 3, 4]. However, there has been a lack of deep analysis on general community pattern networks.

Our work is not limited to a particular network (e.g. a P2P network, or social networks, or web forums); the purpose of this work is to answer the following two questions:

- In what situation can community networks maintain stability with respect to their member numbers?
- Are incentives effective in helping the community network to achieve a stable state?

We have analyzed the requirements to form a stable state in community networks, and will discuss the validity of incentive policies when they are used to enable the cooperation.

The contributions of this paper have been broken down into three categories:

- We propose a new model to describe the stability of the community networks;
- We provide a situation demonstrating the stable state of community networks;
- We extend the model to analyze the incentive effectiveness of different policies.

The organization of this paper is as follows: Discussion of related work is given in Section 2. Section 3 provides the basic of our proposed static game model. We extend this model to repeat the game in Section 4. Then we incorporate incentives to the model in Section 5. Simulation results are given in Section 6, and finally Section 7 concludes this paper.

## Related Works

Normally, a community is stable when its members have no motivation to leave and outside users are not interested in joining the community. The general analysis of stability as stated by D’Aspremont [5] is widely used in research on cooperation in a community’s stability. Recently some approaches like [6, 7] focus mostly on cooperation in a community's stability for a particular type of network, like P2P networks and social networks.

P2P networks introduce incentive mechanisms to maintain the long-term cooperation between members. Incentive policies become the basis of the resource sharing within the networks, in the form of reputation, punishment and price policies. A typical policy is Tit-for-Tat [8, 9] and its extensive versions [10, 11]. The key idea of Tit-for-Tat is to punish the user who refuses to cooperate in the past time. Game theory is widely used to model these types of problems and to analysis different types of incentive policies.

Some research works have introduced service quality as a reward for the services or resources it provides, as in [12] and [13]. The research in [12] uses a score-system with “Points” as the score could be consumed when a member receives the services. The member receives points when they provide data and charge them when they download data. The research in [13] assumes the member is completely rational and proposes a service differential incentive policy. The member who provides more services receives better data sources and increased download bandwidth.

The P2P Community is a new network form for improved data sharing. The research in [14] shows that proportionally shared data as a reward for members' contributions ensures the success of private BitTorrent communities. The work in [15] has theoretically analyzed the incentive policy in P2P community networks, and has proven it is an effective way to promote cooperation, because the membership system can track the user behaviors, which could not be easily performed in open P2P networks. Research performed in [16, 17] describe the ecosystem and user behavior characteristics in P2P communities.

All of the above research has focused on the cooperation in special network forms, which includes more detail on different network environments. In this paper, we've placed our focus on the stability of generalized community networks without restrictions to any special networks.

## Basic Cooperation Model of Community Networks

It is well known that game theory plays a critical role in almost all the scientific disciplines [18, 19, 20, 21, 22]. Many pieces of research have used the dilemma model to promote cooperation [23, 24]. In typical prisoner’s dilemma model, each player has two choices: to contribute to the common good or to shirk to get a free ride on the work of others [25]. In this paper, we used this model to establish a basic analysis model and then extended it to more detailed situations. Here we call the two kinds of players “legal users” and “free riders” respectively. In prisoner’s dilemma model, once the gains of free riders exceed that of legal users, legal users tend to become free riders. This behavior influences the way the community network runs.

We call the inside users “legal users,” part of the outside users “free riders,” and the remaining users “uninterested users.” Legal users share their content (upload) and obtain others’ content (download); free riders only download content. We refer to both legal users and free riders as “interested users”. Free riders are free to decide to join the community and become legal users. Uninterested nodes have nothing to do with the community.

In general, a community network is excellent with more interested users and more content. Here are the assumptions of this paper.

- There are a certain number of users inside the community networks, the number of outside users is at least several orders of magnitude larger than the community’s population.
- Legal users provide all of the contents in the community networks.
- Each user is completely rational.
- Each user has an equal right to access content and can calculate his benefit and make an optimal choice.

### A. A Simple Basic Model

We've used *s = {1*,*2*,*3*,*…*,*n}* as set of legal users, each user *i* (*i* ∈ *s*) possesses a non-negative upload contents *k*_*i*_. *K* represents the amount of content in the community network, *K* = ∑_*n*_ *k*_*i*_. *N* represents the number of interested users, the number of free riders is *N-n*.

Next, we define the utility functions for each type of users. In this paper, we use term “utility” as the gains of a game player. We divided users’ ultimate utility into two parts: gains from downloading content and losses due to the content they have shared.

According to common practices and economic knowledge, the first part of the utility function is a concave function [26]. The physical meaning is the amount of content that is valuable to users out of the rest of the content. According to the economic theory in [9], the outcome of the model is independent of the concrete form of the beneficial function. Without a loss of generality, we used a power function like $y=\sqrt{x}$ as the beneficial function from content downloads. Legal users benefit from downloading content uploaded by other users. Free riders benefit from downloading content provided by all users. Benefits functions of legal user *i* and free riders are *W*_*i*_ and *W’* respectively.

$$W_{i}=a\sqrt{K_{-i}}$$ (1)

$$W\prime =a\sqrt{K}$$ (2)

*K*_−*i*_ = *K* − *k*_*i*_, *a* (*a* > 0) represents the degree of benefits users get from specific resources.

Loss function in the remaining part of the utility function. For legal users, the loss is caused by the effort of uploading content. In general, the revenue function is a concave function, and the cost function is a convex function. Thus, the payoff function is a concave function, in economic, which means the law of diminishing marginal return [27, 28]. In this paper, we use a quadratic function like *y* = *x* ^2^ as legal users’ loss function. Loss function of legal user *i* is

$$E_{i}=-\text{c}k_{i}^{2}$$ (3)

*c* (*c* > 0) represents the degree of users’ loss caused by uploading.

In the community, free riders use illegal methods to download content. For each unit of content they get, they bear a very little loss. We assume for a specified unit of content they will bear 1-*f* (*f* ∈ [0,1]) unit loss. *f* is difficulty factor, which represents the degree of difficulty to obtain the content. For free riders, the less difficult to obtain the content, the larger *f* is. Free riders need *W’* units of content, so loss function is

E′=−(1−f)W′ (4)

At this point, we can obtain both legal users’ and free riders’ utility gain function, *S*_*i*_ and *S’* respectively.

$$S_{i}=W_{i}+E_{i}=a\sqrt{K_{-i}}-ck_{i}^{2}$$ (5)

And let *S’* represents the ultimate gains of the free riders, and then we have

$$S\prime =W\prime +E\prime =f\;a\sqrt{K}$$ (6)

We can conclude the best gains in any specified situation as follows, in any of which the constant *a* and *c* are assigned a value.

Consider maximum of *S*_*i*_: max[$a\sqrt{K_{-i}}-ck_{i}^{2}$]. Each user chooses his upload amount at the same time. Take the first derivative of *S*_*i*_, $S_{i}^{\prime }=\frac{1}{2}a\sqrt{\frac{n-1}{k_{i}}}-2ck_{i}$, let $S_{i}^{\prime }=0$. When $k_{i}=\sqrt[{3}]{\frac{a^{2}\left(n-1\right)}{16c^{2}}}$, *S*_*i*_ is maximum.

$$K=\sqrt[{3}]{\frac{a^{2}\left(n-1\right)n^{3}}{16c^{2}}}$$ (7)

*S*_*i*_(*n*) represents the maximum gains of legal user *i*; *S*′(*n*) represents the maximum gains of free riders when the community population is *n*.

$$S_{i}\left(n\right)=3{\left(\frac{1}{2}\right)}^{\frac{8}{3}}a^{\frac{4}{3}}c^{-\frac{1}{3}}{\left(n-1\right)}^{\frac{2}{3}}$$ (8)

$$S\prime \left(n\right)={\left(\frac{1}{2}\right)}^{\frac{2}{3}}fa^{\frac{4}{3}}c^{-\frac{1}{3}}{\left(n-1\right)}^{\frac{1}{6}}n^{\frac{1}{2}}$$ (9)

In this model, all the players are interested users (legal users and free riders). For each player, strategies are joining or quitting the group of legal users. In each round, the player is aware of the number of both legal users and free riders. Player calculates his best gain at the same time and choose the best strategies. **Table 1** shows the payoff matrix of legal users and free riders. According to (7), both users’ gain and overall contents increase with community population. **S1 Fig** shows the maximum gains of legal users and free riders against different community population with different *f*. Both of legal users and free riders’ optimal gains increase with the growth of community’s population. Free riders’ gain exceeds the legal users’ when *f* = 0.95, whereas, legal users’ gain exceeds the free riders’ when *f* = 0.25 and *f* = 0.50 respectively.

**Table 1:** Payoff matrix of the game.

	Join	Quit
**Join**	*S*_*i*_(*n* + 1)/*S*_*i*_(*n* + 1)	*S*_*i*_(*n*)/*S*′(*n*)
**Quit**	*S*′(*n*)/*S*_*i*_(*n*)	*S*′(*n* − 1)/*S*′(*n* − 1)

The first row and the first line represents legal users’ and free riders’ behavior respectively. A/B, A represents the payoff of legal users; B represents the payoff of free riders.

### B. Stability Analysis of the Basic Model

In this section, we will find the basic model of the cooperation game with a static model and then discuss the stability in these types of community networks. Although more legal users make both legal users and free riders benefit more, is the community network stable with a larger population? We will answer the question of whether the community network will become more stable with more legal users next.

Model in 1 divides stability into internal stability and external stability. Internal stability represents members in the community have no motivation to drop out. External Stability represents outside members have no motivation to join the community.

There are many common situations between community networks and Cartel Model 1, so we've introduced several definitions similar to those in Cartel Model, and draw the conclusions from our model.

Normally, there are three forms of community networks:

- Attracting free riders to join legal users, called attractive community networks;
- Leading legal users becoming free riders, called diseased community networks;
- Both legal users and free riders remain their original status, called stable community networks.

At this point, it is better to introduce more definitions for further analyzing internal and external stability situation.

**Definition 1**: A community network is stable iff (if and only if) it is internal stable and external stable.

**Definition 2**: A community network is attractive iff it is internal stable and is not external stable.

**Definition 3**: A community network is diseased iff it is external stable and is not internal stable.

**Definition 4**: A community network is stable iff there exists a stable interval (with both internal stability and external stability).

**Definition 5**: If the number of legal users can adjust to stable interval spontaneously, the community network is self -adjusting.

**Theorem 1**: If a community network is stable, it’s self-adjusting.

**Proof**. When community population *n* is less than the stable interval’s lower boundary, the community is not external stable, but it is internal stable. Free riders have the motivation to defect. With the idea of the evolutionary game theory, it comes to equilibrium by a process of trial and error [29, 30]. A random number of free riders will join legal users until *n* exceeds upper boundary. Inversely, when *n* is larger than the upper boundary, random legal users will betray the community and join the opposite side until *n* is less than the lower boundary. That is a process of trial and error [31], before *n* eventually stops in the stable interval, the oscillations of *n* get smaller around the interval. The process may take some time, to simplify the calculation, we assume *n* would reach the stable interval in only one round game.

**Theorem 2**: A community network is attractive, legal users’ gains and *K* reach the theoretical maximum.

**Proof.** If the community network is attractive, it is internal stable and is not external stable. Free riders always have the motivation to join legal users. Thus, community population rises to *N*. Gains of legal users and *K* reach the theoretical maximum, *S*_*i*_(*N*) and *K*_*max*_ respectively. We regard the stable interval is *N*.

When a community network is internally stable, legal users will notice that joining free riders could not bring them extra benefit. External stability brings free riders no motivation to join legal users. In this model,

- *S*_*i*_(*n*) ≥ *S*’(*n* − 1), it is internal stable.
- *S*_*i*_(*n* + 1) ≤ *S*’(*n*), it is external stable.

Then we analyze community network’s stability. First, consider internal stability. We can give internal stability condition

$$S_{i}\left(n\right)\geq \;S’\left(n-1\right)$$ (10)

Substitute (8) and (9), we have

$$3{\left(\frac{1}{2}\right)}^{\frac{8}{3}}a^{\frac{4}{3}}c^{-\frac{1}{3}}{\left(n-1\right)}^{\frac{2}{3}}\geq {\left(\frac{1}{2}\right)}^{\frac{2}{3}}fa^{\frac{4}{3}}c^{-\frac{1}{3}}{\left(n-2\right)}^{\frac{1}{6}}{\left(n-1\right)}^{\frac{1}{2}}$$ (11)

Let $\Delta_{1}={\left(\frac{n-1}{n-2}\right)}^{\frac{1}{6}}$, Δ_1_ is extremely approximate to 1 (*n* = 10000, Δ_1_ = 1.000017; *n* = 1000000, Δ_1_ = 1.000001). When *f* ≤ 0.75Δ_1_, inequality (11) is satisfied, and the community network is internal stable; Otherwise, it is not internal stable.

External stability condition

$$S_{i}\left(n+1\right)\leq S’\left(n\right)$$ (12)

Substitute (8) and (9), we have

$$3{\left(\frac{1}{2}\right)}^{\frac{8}{3}}a^{\frac{4}{3}}c^{-\frac{1}{3}}n^{\frac{2}{3}}\leq {\left(\frac{1}{2}\right)}^{\frac{2}{3}}fa^{\frac{4}{3}}c^{-\frac{1}{3}}{\left(n-1\right)}^{\frac{1}{6}}n^{\frac{1}{2}}$$ (13)

Let $\Delta_{2}={\left(\frac{n}{n-1}\right)}^{\frac{1}{6}}$ (Δ_2_ < Δ_1_). When *f* ≥ 0.75Δ_2_, inequality (12) is satisfied, and the community network is external stable; Otherwise, it is not external stable. Property of the community network is shown in **Table 2**.

**Table 2:** Stability of community network in basic model.

***f***	***f* < 0.75Δ_2_**	**0.75Δ_2_ ≤ *f* ≤ 0.75Δ_1_**	***f* > 0.75Δ_1_**
**Community network property**	*attractive*	*stable*	*diseased*

When 0.75Δ_2_ ≤ *f* ≤ 0.75Δ_1_, the community network is stable, and stable interval is $\left[1+\frac{1}{X-1},2+\frac{1}{X-1}\right],\;(X={\left(\frac{f}{0.75}\right)}^{6}$).

## Repeat Game Model on Community Networks

We’ve found that both legal users and free riders gain more when the number of legal users is larger. However, in some cases a one-stage game cannot increase community population, and the condition of *N* legal users is not stable. But in a repeated game, the stable interval may change which was first proved in a general case [32]: In repeated indefinite games, if participates have enough patience, the outcome can be supported by a sub-game perfect equilibrium.

In a repeated game, if free riders find out joining legal users is a better choice in the long term, they will sacrifice short-term interests and join legal users for long-term interests. The outcome that is not supported by a static game will be supported by a dynamic repeated game. In this section, we’ll build a repeated game model to find new stable interval supported by the sub-game perfect equilibrium. In this model, the one-stage game is one round and repeatedly executes. Additionally, there exists unique Nash equilibrium (stable interval) in a one-stage game.

At the new stable interval, once a legal user betrays, other legal users will notice this phenomenon. In the next stage, the community networks will go back to the one-stage game’s stable interval and maintain that state to the end. For legal users, maintaining their identity is a better choice if one stage’s benefit could not balance the loss after betraying. When the discount factor is large enough, and participants are patient enough, it is worth staying with legal users for long-term interests.

Next part of this section will find the new stable interval. We must first assume the new stable interval exists and is close to *h* (*h* ≤ *N*).

**Theorem 3**: If the discount factor is large enough, there exists *h*_*0*_, we have *h* ≥ *h*_0_.

**Proof.** Since users are same, their discount factors δ ∈ (0,1) are same too. To simplify the calculation, we must ignore the triggered process. That is, once a legal user drops out, next stage the community networks will revert to the primary stable interval.

When *f* ≤ 0.75Δ_2_, the community network is attractive. Gains of free riders are always less than that of legal users, so free riders will join legal users with no doubt. Thus, the new stable interval is *N*, *h*_0_ = *N*.

When 0.75Δ_2_ ≤ *f* ≤ 0.75Δ_1_, assume primary stable interval is [*n*_1_, *n*_2_], let *n*_0_ ∈ [*n*_1_, *n*_2_]. If legal users would maintain the new stable interval, they must meet the following condition

$$\begin{array}{c} S_{i}\left(h\right)+\delta S_{i}\left(h\right)+\delta^{2}S_{i}\left(h\right)+\cdots +\delta^{n}S_{i}\left(h\right)\geq \\ S’\left(h-1\right)+\;\delta \;S’\left(n_{0}\right)+\delta^{2}\;S’\left(n_{0}\right)+\cdots +\delta^{n}\;S’\left(n_{0}\right) \end{array}$$ (14)

Simplified to:

$$\frac{1}{1-\delta }S_{i}\left(h\right)\geq \text{S}\prime \left(\text{h}-1\right)+\frac{\delta }{1-\delta }S’\left(n_{0}\right)$$ (15)

Let $\varepsilon ={\left(h-1\right)}^{\frac{2}{3}}-{\left(h-2\right)}^{\frac{1}{6}}{\left(h-1\right)}^{\frac{1}{2}}$, (15) simplified to

$${\left(h-1\right)}^{\frac{2}{3}}\geq \left[\frac{\delta f{\left(n_{0}-1\right)}^{\frac{1}{6}}n_{0}{}^{\frac{1}{2}}-\left(1-\delta \right)f\varepsilon }{0.75-\left(1-\delta \right)f}\right]$$ (16)

Generally, *δ* is closely approximate to 1 (0.9999).

Let $h_{0}=\left[\frac{\delta f{\left(n_{0}-1\right)}^{\frac{1}{6}}n_{0}{}^{\frac{1}{2}}-\left(1-\delta \right)f\varepsilon }{0.75-\left(1-\delta \right)f}\right]{\;}^{\frac{3}{2}}+1$. When *f* > 0.75Δ_1_, community network is diseased, and the condition is similar to (16), but stable interval doesn’t exist. Thus, *n*_*0*_ could be extremely tiny. When *h* > *h*_0_, legal users have no motivation to drop out.

**S2 Fig** indicates legal users’ gains with different behavior. The imaginary line represents the gain that legal user does not betray, and the solid line represents the gain if he chooses to be a free rider. If he betrays, he will immediately get much more gains than what he gets if he chooses to remain, namely short-term gains. However, for a long period his gains is a little bit less than that if he chooses to remain, namely long-term lost. The area of two gray cross sections corresponds to the value of short-term gains and long-term lost respectively. Legal users will stay in their original block if long-term lost exceed short-term gains; otherwise they will betray. At present, we have ranges of the new stable interval, but to determine the value we need consider free riders’ strategy.

**Theorem 4**: In repeated game, there exists *h*_*1*_, and when *n* > *h*_1_, free riders have the motivation to join legal users.

**Proof.** *n*_*0*_ represents the initial number of legal users. Once a free rider joins in, the next stage of the community networks will move to the new stable interval. We must ignore the triggered process too. The new stable interval should meet the following conditions

$$\begin{array}{l} \;S\prime \left(n_{0}\right)+\delta S\prime \left(n_{0}\right)+\delta^{2}S\prime \left(n_{0}\right)+\cdots +\delta^{n}S\prime \left(n_{0}\right)<\\ S_{i}\left(n_{0}+1\right)+\delta S_{i}\left(h\right)+\delta^{2}S_{i}\left(h\right)+\cdots +\delta^{n}S_{i}\left(h\right) \end{array}$$ (17)

Simplified to

$$\frac{1}{1-\delta }S\prime \left(n_{0}\right)<S_{i}\left(n_{0}+1\right)+\frac{\delta }{1-\delta }S_{i}\left(h\right)$$ (18)

Let $\varepsilon =n_{0}{}^{\frac{2}{3}}-{\left(s_{0}-1\right)}^{\frac{1}{6}}n_{0}{}^{\frac{1}{2}}$, (18) simplified to

$${\left(h-1\right)}^{\frac{2}{3}}\geq \left[\frac{\left[f-0.75\left(1-\delta \right)\right]{\left(n_{0}-1\right)}^{\frac{1}{6}}n_{0}{}^{\frac{1}{2}}+0.75\left(1-\delta \right)\varepsilon }{0.75\delta }\right]$$ (19)

Let $h_{1}\;={\left[\frac{\left[f-0.75\left(1-\delta \right)\right]{\left(n_{0}-1\right)}^{\frac{1}{6}}n_{0}{}^{\frac{1}{2}}+0.75\left(1-\delta \right)\varepsilon }{0.75\delta }\right]}^{\frac{3}{2}}+1$, When *n* > *h*_1_, inequality (19) is satisfied, and free riders have the motivation to join legal users.

**S3 Fig** shows free riders’ gains with different behavior. Similar to **S2 Fig**, the imaginary line represents the gain that free rider chooses to join legal users, and the solid line represents the gain if he chooses to remain. If he chooses to be a legal user, he will immediately bear a loss compared with what he gets if he chooses to remain, namely short-term lost. However, his gains is a little bit more than that if he chooses to remain for a long period, namely long-term gains. The area of two gray cross sections corresponds to the value of short-term lost and long-term gains respectively. Free riders will stay in their original block if short-term lost exceed long-term gains; otherwise they will betray.

**Theorem 5**: In a repeated game, if *δ* is large enough, *N* is the new stable interval.

**Proof.** If *f* ≤ 0.75Δ_2_, community network is attractive. Gains of free riders are always less than that of legal users, so free riders will join legal users with no doubt. Thus, the new stable interval is *N*.

If *f* > 0.75Δ_2_, *h*_*1*_ and *h*_*2*_ both extremely close to the original stable interval. Free riders find themselves able to make legal users lose the motivation to drop out after they have joined the community. As a result, they are willing to join legal users to improve their benefits and the number of legal users will increase up to *N*. Thus, the new stable point is *N*.

If users are patient enough, they will value long-term gains and be willing to become legal users. However, we ignored the triggered process. If we consider the triggered process, the cost of dropout becomes larger, and users should be more patient to balance the dropout benefits.

## Advanced Model with Cooperation Incentives

The basic model can reveal the stability conditions of community networks with simple policies. The original game model without incentives makes it harder to maintain cooperation between members. To attract prospective members to join the community, incentive policies [33, 34, 35] have been proposed by many community networks. In this section, we introduce an advanced model with score systems as incentive method.

In this model, legal users need scores to download content. Like the basic model, free riders must expend extra effort to get the content they need. Except for gains from downloading content, legal users will earn reward via scores by uploading content. The reward differs from the actual gains; the reward scores are determined by content user upload and the downloading of others’ content.

### A. Model with Score System

Most websites use the linear function as reward function [36, 37], like Bulletin Board System (BBS). We assume all content is of equal value in this model. *R*_*i*_ represents the reward function; *d* (*d* > 0) is reward factor, larger *d* represents stronger incentive.

$$R_{i}=dk_{i}$$ (20)

In this model, we've taken into consideration when one’s scores are too high to benefit maximumly from them. We unify the benefits function and the reward function. *L*_*i*_ represents the benefit function, and *L*_*i*_ = min[*R*_*i*_, *W*_*i*_].

Then we have their ultimate utility function. *S*_*i*_, *S’* represents the ultimate gains of legal user *i* and free riders respectively.

$$S_{i}=L_{i}+E_{i}=L_{i}-ck_{i}^{2}$$ (21)

$$S\prime =W\prime +E\prime =fa\sqrt{K}$$ (22)

Then we can conclude the best gains for both legal user and free riders.

Let $S_{i}^{*}=dk_{i}-ck_{i}^{2}$, take the first derivative of *S*_i_^*^. $k_{0}=\frac{d}{2c}$, *S*_i_^*^ is maximized.

When $n\geq \frac{d^{3}}{2a^{2}c}-1,\;k_{i}=\frac{d}{2c}$, *S*_*i*_ is maximized.

$$S_{i}\left(n\right)=\frac{d^{2}}{4c}$$ (23)

$$S\prime \left(n\right)=fa\sqrt{\frac{nd}{2c}}$$ (24)

When $n<\frac{d^{3}}{2a^{2}c}-1,\;k_{i}=\frac{a^{2}\left(n-1\right)}{d^{2}}$, *S*_*i*_ is maximized.

$$S_{i}\left(n\right)=\frac{a^{2}\left(n-1\right)}{d}-\frac{a^{4}c\left(n-1\right)}{d^{4}}$$ (25)

$$S\prime \left(n\right)=\frac{fa^{2}\sqrt{\left(n-1\right)n}}{d}$$ (26)

### B. Stability Analysis

In this section, we will analyze the stability of community networks with the score system. In different ranges, *S*_*i*_(*n*) and *S*′(*n*) differ, so we will discuss them separately.

- $n<\frac{d^{3}}{2a^{2}c}+1$

Same to the basic model, we provide internal stability condition

$$S_{i}\left(n\right)\geq \;S’\left(n-1\right)$$ (27)

Substitute (25) and (26), we have

$$\left(\frac{a^{2}}{d}-\frac{a^{4}c}{d^{4}}\right)\left(n-1\right)-\frac{fa^{2}\sqrt{\left(n-2\right)\left(n-1\right)}}{d}\geq 0$$ (28)

Let $\Delta_{3}={\left(\frac{n-1}{n-2}\right)}^{\frac{1}{2}}$. If $\frac{f}{1-\frac{a^{2}c}{d^{3}}}\leq \Delta_{3}$, inequality (28) is satisfied and the community network is internal stable. Otherwise, it is not internal stable.

Here we give external stability condition similar to basic model

$$S_{i}\left(n+1\right)\leq S’\left(n\right)$$ (29)

Substitute (25) and (26), we have

$$\left(\frac{a^{2}}{d}-\frac{a^{4}c}{d^{4}}\right)n-\frac{fa^{2}\sqrt{\left(n-1\right)n}}{d}\leq 0$$ (30)

Let $\Delta_{4}={\left(\frac{n}{n-1}\right)}^{\frac{1}{2}}\;(\Delta_{4}<\;\Delta_{3}$). If $\frac{f}{1-\frac{a^{2}c}{d^{3}}}\geq \Delta_{4}$, inequality (30) is satisfied, and the community network is external stable. Otherwise, it is not external stable.

Stability of community network is shown in

**Table 3**. When $\Delta_{4}\leq \frac{f}{1-\frac{a^{2}c}{d^{3}}}\leq \Delta_{3}$, the community network is stable. In addition, if ${\left(\frac{f}{1-\frac{a^{2}c}{d^{3}}}\right)}^{2}<\frac{2a^{2}c}{d^{3}}+1,\;1+\frac{1}{X-1}>\frac{d^{3}}{2a^{2}c}+1$ and stable interval is out of range. Otherwise stable interval is:

$$\left[1+\frac{1}{X-1},\text{min}(2+\frac{1}{X-1},\frac{d^{3}}{2a^{2}c}+1)\right],\;(X={\left(\frac{f}{1-\frac{a^{2}c}{d^{3}}}\right)}^{2}).$$

**Table 3:** Stability of community network in case 1.

***f***	$\frac{f}{1-\frac{a^{2}c}{d^{3}}}<\Delta_{4}$	$\Delta_{4}\leq \frac{f}{1-\frac{a^{2}c}{d^{3}}}\leq \Delta_{3}$	$\frac{f}{1-\frac{a^{2}c}{d^{3}}}\geq \Delta_{3}$
**Community network property**	*Attractive*	*Stable*	*Diseased*

- $n>\frac{d^{3}}{2a^{2}c}+1$

Substitute (23) and (24) to (27). Internal stability condition turns to be

$$\frac{d^{2}}{4c}-fa\sqrt{\left(n-1\right)\frac{d}{2c}}\geq 0$$ (31)

If *f* ≤ 0.5 and $n<\frac{d^{3}}{8a^{2}cf^{2}}+1$, inequality (31) is satisfied and the community network is internal stable. Otherwise, it is not internal stable.

Substitute (23) and (24) to (29). External stability condition turns to be

$$\frac{d^{2}}{4c}-fa\sqrt{\frac{nd}{2c}}\leq 0$$ (32)

If $n\geq \frac{d^{3}}{8a^{2}cf^{2}}$, inequality (32) is satisfied, and the community network is external stable. Otherwise, it is not external stable.

When *f* ≤ 0.5, the community network is stable. In addition, if $f>0.5,\;\frac{d^{3}}{8a^{2}cf^{2}}+1<\frac{d^{3}}{2a^{2}c}+1$ and stable interval does not exist. Otherwise stable interval is $\left[\frac{d^{3}}{8a^{2}cf^{2}}\;,\;\frac{d^{3}}{8a^{2}cf^{2}}+1\right]$.

- $n=\frac{d^{3}}{2a^{2}c}+1$

Substitute (23) and (26) to (27). Internal stability condition turns to be

$$\frac{d^{2}}{4c}-\frac{fa^{2}\sqrt{\left(n-2\right)\left(n-1\right)}}{d}\geq 0$$ (33)

If $f\leq \frac{1}{2\sqrt{1-\frac{2a^{2}c}{d^{3}}}}$, inequality (33) is satisfied and the community network is internal stable. Otherwise, it is not internal stable.

Substitute (23) and (26) to (29). External stability condition turns to be

$$\frac{d^{2}}{4c}-fa\sqrt{\frac{nd}{2c}}\leq 0$$ (34)

If $f\geq \frac{1}{2\sqrt{1+\frac{2a^{2}c}{d^{3}}}}$, inequality (34) is satisfied and the community network is external stable. Otherwise, it is not external stable. When $\frac{1}{2\sqrt{1+\frac{2a^{2}c}{d^{3}}}}\leq f\leq \frac{1}{2\sqrt{1-\frac{2a^{2}c}{d^{3}}}}\;,\;n=\frac{d^{3}}{2a^{2}c}+1$ is stable interval.

**Theorem 6**: In incentive game model, there exists unique stable interval.

**Proof.** In case 1, if stable interval exists, it must meet ${\left(\frac{f}{1-\frac{a^{2}c}{d^{3}}}\right)}^{2}\geq \frac{2a^{2}c}{d^{3}}+1$ and $\Delta_{4}\leq \frac{f}{1-\frac{a^{2}c}{d^{3}}}\leq \Delta_{3}$. Let Δ_4_ = 1 + *ε* (*ε* → 0^+^), we have $d^{3}\geq \frac{2a^{2}c}{\varepsilon^{2}+2\varepsilon }$. Thus, *d^3^* is much larger than *2a^2^c*, and *f* should be close to $\left(1-\frac{a^{2}c}{d^{3}}\right)$. In case 2, if stable interval exists, *f* ≤ 0.5. That conflicts with case 1 which means case 1 and case 2 cannot have stable interval all at once.

Stability of community network is shown in **Table 4**. When $\Delta_{4}\leq \frac{f}{1-\frac{a^{2}c}{d^{3}}}\leq \Delta_{3}$, the community network is stable. In addition, if ${\left(\frac{f}{1-\frac{a^{2}c}{d^{3}}}\right)}^{2}<\frac{2a^{2}c}{d^{3}}+1,\;1+\frac{1}{X-1}>\frac{d^{3}}{2a^{2}c}+1$ and stable interval does not exist. Otherwise stable interval is:

$$\left[1+\frac{1}{X-1},\text{min}(2+\frac{1}{X-1},\frac{d^{3}}{2a^{2}c}+1)\right],\;(X={\left(\frac{f}{1-\frac{a^{2}c}{d^{3}}}\right)}^{2}).$$

**Table 4:** Stability of community network in advanced model.

***f***	*f* ≤ 0.5	*f* > 0.5		
***d***		$\frac{2a^{2}c\Delta_{4}}{\Delta_{4}-f}>d^{3}>\frac{2a^{2}c\Delta_{3}}{\Delta_{3}-f}$ & $d^{3}\geq \frac{2a^{2}c}{\varepsilon^{2}+2\varepsilon }$	$d^{3}>\frac{2a^{2}c\Delta_{3}}{\Delta_{3}-f}$ & $f\leq \frac{1}{2\sqrt{1-\frac{2a^{2}c}{d^{3}}}}$	$d^{3}<\frac{2a^{2}c\Delta_{3}}{\Delta_{3}-f}$
**Community network property**	*Stable*	*Stable*	*Stable*	*Diseased*
**Stable interval**	$\left[\frac{d^{3}}{8a^{2}cf^{2}}\;,\;\frac{d^{3}}{8a^{2}cf^{2}}+1\right]$	$\left[\begin{array}{c} 1+\frac{1}{X-1},\\ \min(2+\frac{1}{X-1},\frac{d^{3}}{2a^{2}c}+1) \end{array}\right]$, ($X={\left(\frac{f}{1-\frac{a^{2}c}{d^{3}}}\right)}^{2}$).	$\frac{d^{3}}{2a^{2}c}+1$	

### C. Effectiveness of Incentives

In this model, we will determine whether the value of the motivation factor is influential in the stable interval. **S4 Fig** shows the stable interval’s variation against *d* (*f* = 0.400). It is obvious that increasing *d* can always raise stable internal. It should be noticed that when *f* > 0.5, the community network can hardly get stable or obtain the impressive stable interval.

## Simulation

Research in [38] promote a cooperation considering human interaction. We’ve programmed a simulation tool to verify our models. In our program, each user has a trial-and-error factor to indicate their possibility of betraying, which is similar to human’s behavior. Each user can calculate his maximum gains according to the current state, and choose to betray his original group. Additionally, there are 10,000 interested users in the community and 5,000 initial population. We set other parameters *a* = 0.1000, *c* = 1.0000 in our simulator.

For the basic model, we simulate the population changes with different *f*, which contains all conditions (community network is stable, attractive, diseased). **S5 Fig** shows how the community network population changes for 50 rounds. When *f* is much less than 0.75 (*f* = 0.60000), being legal users is more attractive, and the community network gets to its maximum population (10,000) very fast (in about ten rounds). When *f* is much larger than 0.75 (*f* = 0.90000), it is more cost-effective to join free riders, and the community’s population reaches the minimum quickly. However, when *f* → 0.75^+^ (0.75002 and 0.75003 respectively), the population converges to a stable interval in about 40 rounds. Besides, the stable population is more considerable with larger *f*.

In repeated version of the basic model, we set *δ* = 0.999 and 0.900 respectively to observe the fluctuation of the community’s population, as shown in **S6 Fig**. The Community’s population reaches its maximum (10,000) in a few rounds (population converges faster with larger *δ*), which is supported by the model proposed in Section 4. It makes sense that users tend to be legal users when considering long-term gains with a large *δ*.

In the advanced model simulation (*f* = 0.30000), we take different *d* (3.00 and 3.60 respectively) to simulate the change of population, as shown in **S7 Fig**. The community’s population fluctuates sharply in the first several rounds and converges to a stable interval at about 40 rounds. Consequently, the community attracts more legal users with more incentives.

In the basic model simulation, the ultimate population is 10,000 (*f* = 0.60000), 6,471 (*f* = 0.75002), 3,763 (*f* = 0.75003) and 5 (*f* = 0.90000=) respectively. Theoretical consequence with same parameters (*a* = 0.1000, *c* = 1.0000) is 10,000 (*f* = 0.60000), 6,471 (*f* = 0.75002), 3,763 (*f* = 0.75003) and 5 (*f* = 0.90000) respectively. In repeated version of the basic model simulation, the ultimate population is both 10,000 when *δ* = 0.999 and 0.900, same as theoretical value (10,000). In the advanced model simulation, the ultimate population is 6,471 (*d* = 3.60) and 3,763 (*d* = 3.00) respectively. The outcome in simulation meets the theoretical value (6,471 and 3,763 respectively) perfectly.

## Conclusion

Community networks are widely used on the Internet. The purpose of community networks is to attract more users to join as paying members. The community's policy plays the most important role, even over the content the site provides. In this paper, we provided two kinds of game theory models to describe the actions of sharing contents within the community: a basic model and an advanced model with incentives. And we define three types of community network based on the stability: attractive, stable and diseased. We’ve found that the community network is perfect when there are no free riders. Unfortunately, the static game analysis in the simple model cannot force all free riders join as legal users. However, when we addressed the problem in a long-term repeated game, we found that a perfect community network can be supported by a dynamic repeated game under some conditions. And we found the minimum discount factor.

We proved that it could easily achieve perfect conditions if a community network is designed properly, and it is easy to reach the bad conditions with improper design. Certainly, the community network tends to be perfect under the condition that all users have long prospects, which in classical game theory is called a complete rationality assumption. In the advanced model, a motivation factor will influence the intervals of the stable state. And the repeated game in the advanced model is similar to that in the basic model. Therefore, we didn’t discuss it in this work. However, in reality, it is almost impossible to achieve the best condition or the worst condition due to complex situations.

When we discuss the stability, we have assumed the number of interested users is sufficient. If we remove this assumption, the outcome becomes more complicated; however, it won’t influence the regular pattern. The community network we have discussed in this paper abstracts some common situations from different network forms, like peer-to-peer networks, social networks, even Multiplayer online games. Consequently, the models in this paper will have value on the designs of these types of networks from the points of game theory and stability.

## Supporting Information

S1 Fig Max gains of legal users and free riders with different *f* against community population. (TIF)

S2 Fig Gains of different behavior of legal users for a long period. (TIF)

S3 Fig Gains of different behavior of free riders for a long period. (TIF)

S4 Fig Stable interval against *d* in advanced model. (TIF)

S5 Fig Simulation of the basic model with different *f* for 50 rounds. (TIF)

S6 Fig Simulation of repeated version of the basic model with different *δ* for 50 rounds. (TIF)

S7 Fig Simulation of the advanced model with different *d* for 50 rounds. (TIF)

S1 Data Result of simulation. Simulation result of the basic model and advanced model.
(XLSX)
