# Correction: Game Theoretical Analysis on Cooperation Stability and Incentive Effectiveness in Community Networks

**DOI:** 10.1371/journal.pone.0148688

**Published:** 2016-02-01

**Authors:** Kaida Song, Rui Wang, Yi Liu, Depei Qian, Han Zhang, Jihong Cai

The following information is missing from the Funding section: "This research is supported by 863 Program of China under grant 2012AA010902, by the NSF of China under grant 61202425, by State Key Laboratory of Software Development Environment under grant SKLSDE-2013ZX-22, and by the Fundamental Research Funds for the Central Universities."
