# Supplementary figures and images for: Game Theoretical Analysis on Cooperation Stability and Incentive Effectiveness in Community Networks
Source: PLoS One. 2015 Nov 9;10(11):e0141755. doi: 10.1371/journal.pone.0141755 (PMC4638359; doi:10.1371/journal.pone.0141755)

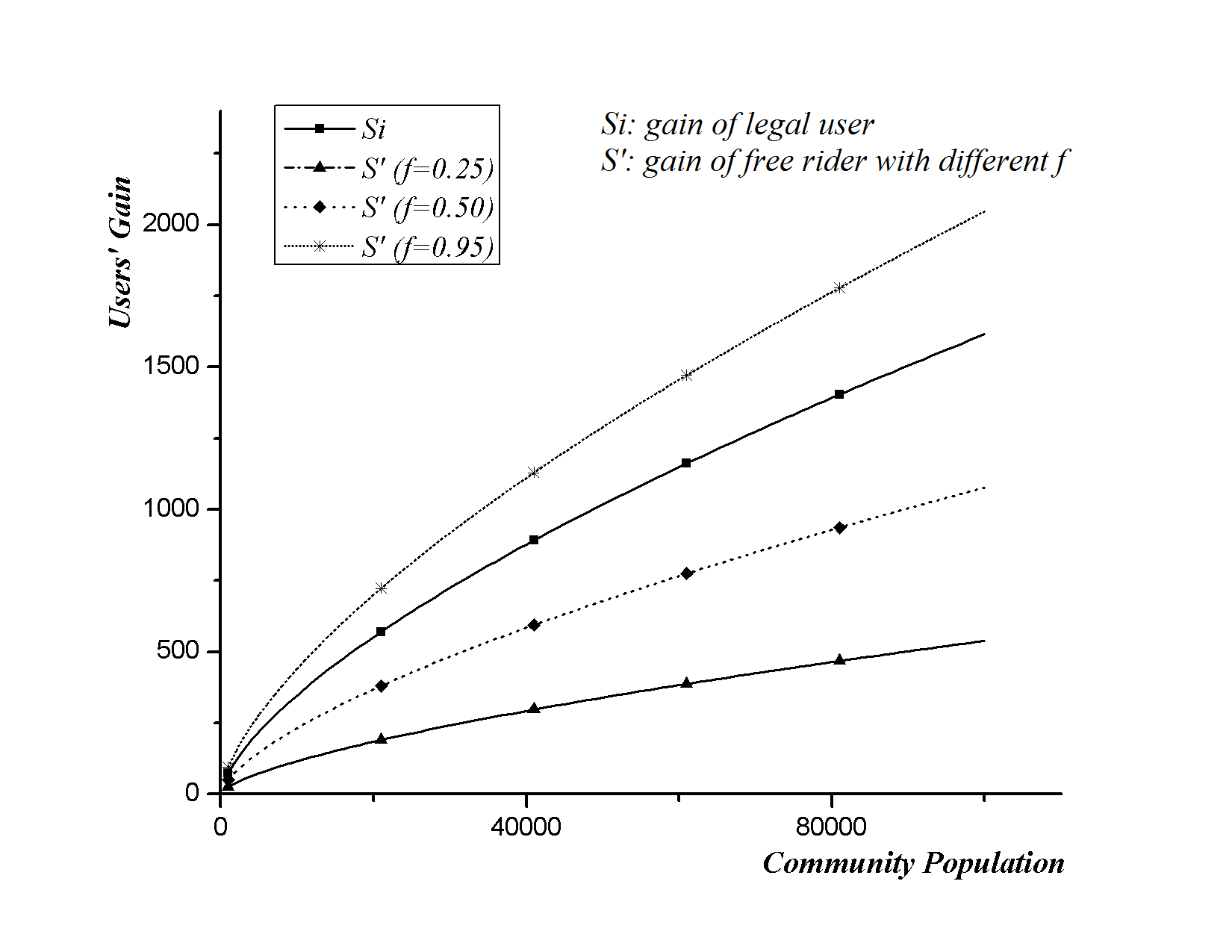

Supplement: S1 Fig — (TIF) [file pone.0141755.s001.tif]

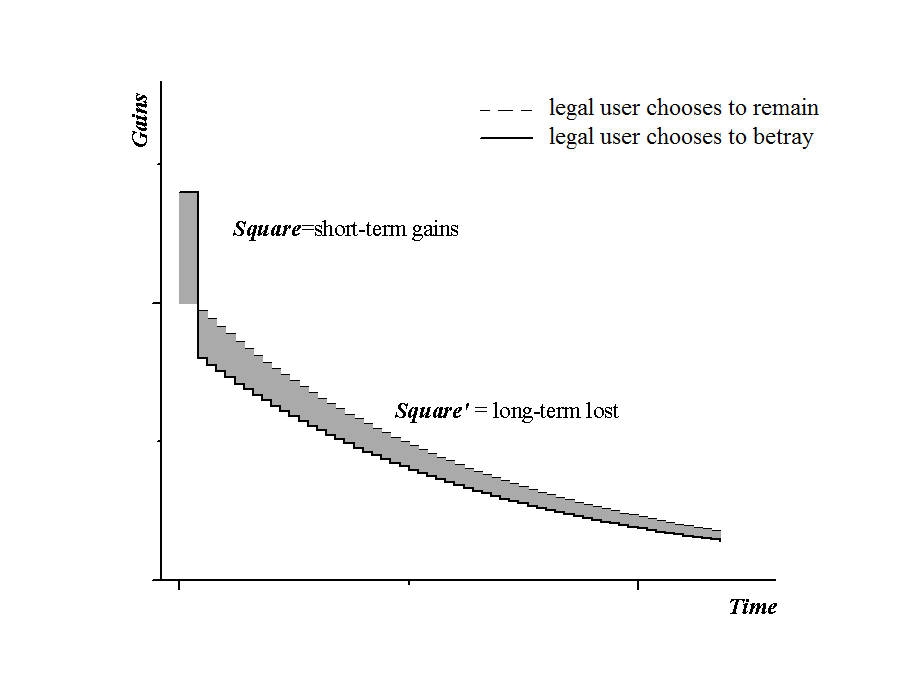

Supplement: S2 Fig — (TIF) [file pone.0141755.s002.tif]

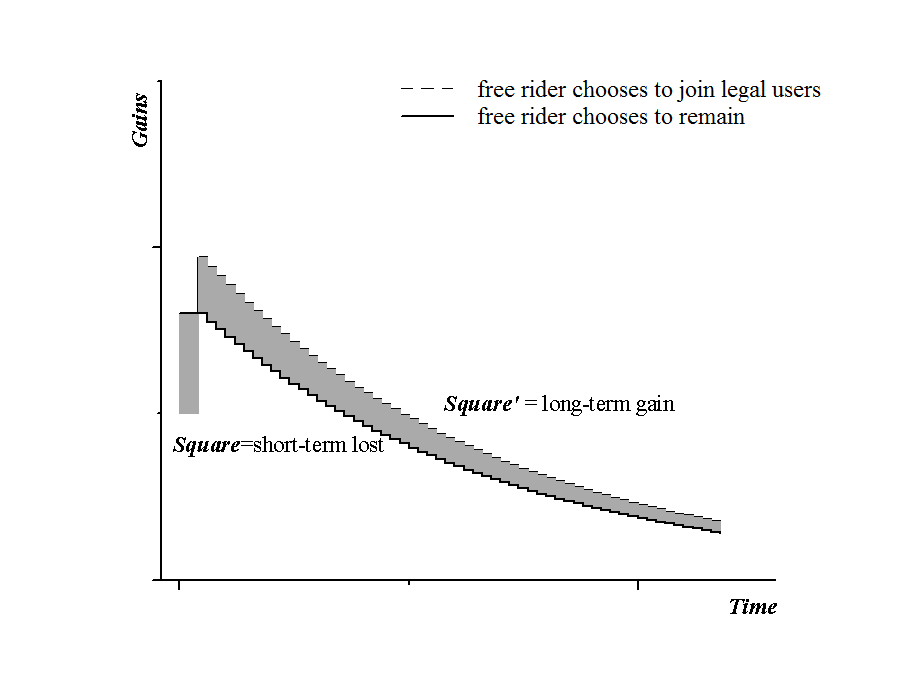

Supplement: S3 Fig — (TIF) [file pone.0141755.s003.tif]

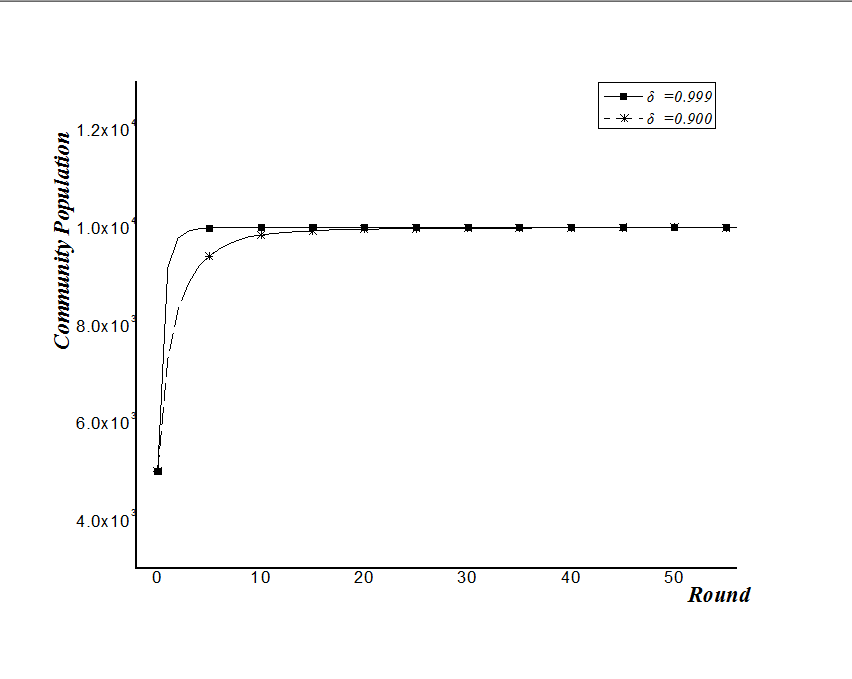

Supplement: S6 Fig — (TIF) [file pone.0141755.s006.tif]

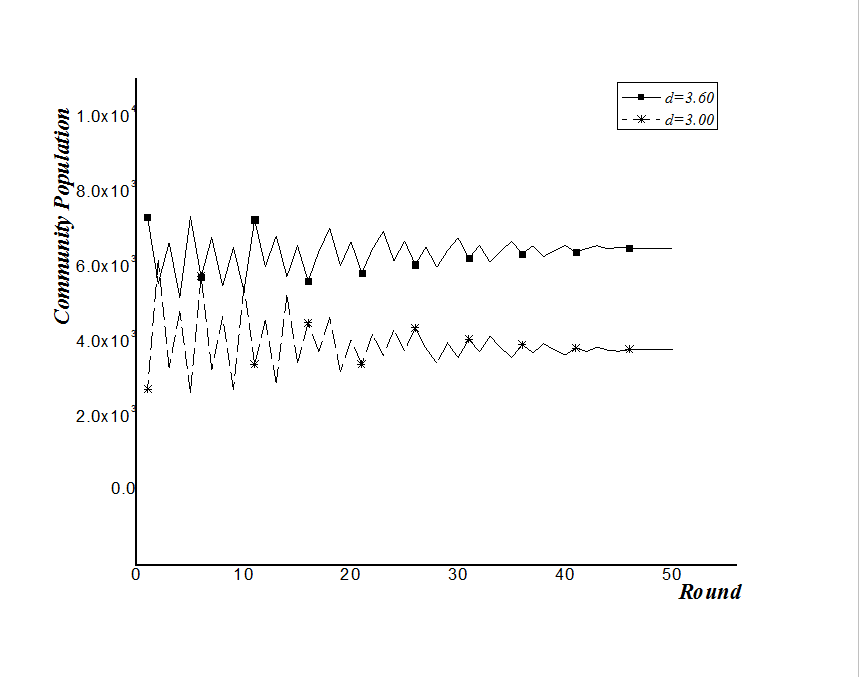

Supplement: S7 Fig — (TIF) [file pone.0141755.s007.tif]
